# In vitro efficacy of ARQ 092, an allosteric AKT inhibitor, on primary fibroblast cells derived from patients with PIK3CA-related overgrowth spectrum (PROS)

**DOI:** 10.1007/s10048-018-0540-1

**Published:** 2018-03-16

**Authors:** C. Ranieri, S. Di Tommaso, D. C. Loconte, V. Grossi, P. Sanese, R. Bagnulo, F. C. Susca, G. Forte, A. Peserico, A. De Luisi, A. Bartuli, A. Selicorni, D. Melis, M. Lerone, A. D. Praticò, G. Abbadessa, Y. Yu, B. Schwartz, Martino Ruggieri, Cristiano Simone, Nicoletta Resta

**Affiliations:** 1 Division of Medical Genetics, Department of Biomedical Sciences and Human Oncology (DIMO), University of Bari “Aldo Moro”, Piazza G. Cesare, 11, Bari, Italy; 2 Medical Genetics, National Institute for Gastroenterology, IRCCS ‘S. de Bellis’, Piazza G. Cesare, 11, Castellana Grotte, Bari, Italy; 3 Unit of Rare Diseases and Medical Genetics, Bambino Gesù Children’s Hospital, Rome, Italy; 4 Unit of Pediatrics, Presidio S. Fermo, ASST Lariana, Como, Italy; 5 Department of Translational Medical Science, Section of Pediatrics, University of Naples Federico II, Naples, Italy; 6 Unit of Medical Genetics, Giannina Gaslini Institute, Genoa, Italy; 7 Unit of Rare Diseases of the Nervous System in Childhood, Department of Clinical and Experimental Medicine, Section of Pediatrics and Child Neuropsychiatry, University of Catania, Via Santa Sofia, 78, 95124 Catania, Italy; 8 Maurice Wohl Clinical Neuroscience Institute, King’s College London, London, UK; 9 Clinical Development, Translational Research, Medical Affairs, ArQule, Inc., Burlington, MA USA; 10 Translational Research, ArQule, Inc., Burlington, MA USA

**Keywords:** PI3K/AKT/mTOR pathway, PI3K/AKT/mTOR inhibitors, PIK3CA mutations, Rapamycin, Wortmannin, Mosaic neurocutaneous disorders, PROS, Target therapy

## Abstract

Postzygotic mutations of the *PIK3CA* [phosphatidylinositol-4,5-bisphosphate 3-kinase catalytic subunit alpha] gene constitutively activate the PI3K/AKT/mTOR pathway in PIK3CA-related overgrowth spectrum (PROS) patients, causing congenital mosaic tissue overgrowth that even multiple surgeries cannot solve. mTOR inhibitors are empirically tested and given for compassionate use in these patients. PROS patients could be ideal candidates for enrolment in trials with PI3K/AKT pathway inhibitors, considering the “clean” cellular setting in which a unique driver, a *PIK3CA* mutation, is present. We aimed to assess the effects of blocking the upstream pathway of mTOR on PROS patient-derived cells by using ARQ 092, a potent, selective, allosteric, and experimental orally bioavailable and highly selective AKT-inhibitor with activity and long-term tolerability, currently under clinical development for treatment of cancer and Proteus syndrome. Cell samples (i.e., primary fibroblasts) were derived from cultured tissues obtained from six PROS patients [3 boys, 3 girls; aged 2 to 17 years] whose spectrum of PIK3A-related overgrowth included HHML [hemihyperplasia multiple lipomatosis; *n* = 1], CLOVES [congenital lipomatosis, overgrowth, vascular malformations, epidermal nevi, spinal/skeletal anomalies, scoliosis; *n* = 1], and MCAP [megalencephaly capillary malformation syndrome; *n* = 4]. We performed the following: (**a**) a deep sequencing assay of PI3K/AKT pathway genes in the six PROS patients’ derived cells to identify the causative mutations and (**b**) a pathway analysis to assess the phosphorylation status of AKT [Ser473 and Thr308] and its downstream targets [pAKTS1 (Thr246), pRPS6 (Ser235/236), and pRPS6Kβ1 (Ser371)]. The anti-proliferative effect of ARQ 092 was tested and compared to other PI3K/AKT/mTOR inhibitors [i.e., wortmannin, LY249002, and rapamycin] in the six PROS patient-derived cells. Using ARQ 092 to target AKT, a critical node connecting PI3K and mTOR pathways, we observed the following: (1) strong anti-proliferative activity [ARQ 092 at 0.5, 1, and 2.5 μM blunted phosphorylation of AKT and its downstream targets (in the presence or absence of serum) and inhibited proliferation after 72 h; rapamycin at 100 nM did not decrease AKT phosphorylation] and (2) less cytotoxicity as compared to rapamycin and wortmannin. We demonstrated the following: (a) that PROS cells are dependent on AKT; (b) the advantage of inhibiting the pathway immediately downstream of PI3K to circumventing problems depending on multiple classes a PI3K kinases; and (c) that PROS patients benefit from inhibition of AKT rather than mTOR. Clinical development of ARQ 092 in PROS patients is on going in these patients.

**Electronic supplementary material:**

The online version of this article (10.1007/s10048-018-0540-1) contains supplementary material, which is available to authorized users.

## Background

*Phosphatidylinositol-4,5-bisphosphate 3-kinase catalytic subunit alpha* (*PIK3CA*; MIM # 171834) gene-related overgrowth (and vascular malformation) syndromes comprise a heterogeneous group of rare, congenital, segmental overgrowth phenotypes underlying somatic activating mutations of genes in the PI3K/AKT/mTOR pathway [1–6]. A wide spectrum of tissues is involved in such abnormal developmental syndromes with increased growth, including brain, blood, fat, skin, vasculatures, and connective tissues [1, 2, 4, 5]. The association of benign and malignant tumors is not common but has been reported [7–9].

The whole spectrum of clinical abnormalities related to *PIK3CA* mutations [1,2,9] is now called *PIK3CA-related overgrowth spectrum* (PROS) and includes the following [1–3, 10, 11]: fibroadipose (and bone) hyperplasia or overgrowth (FAO) [12, 13]; hemihyperplasia multiple lipomatosis (HHML) [11]; type I macrodactyly and muscle hemihypertrophy (HH) [14]; facial infiltrating lipomatosis (FIL) [15]; isolated large lymphatic malformation (ILM) [16, 17]; epidermal nevi (EN), seborrheic keratosis (SK) [18], and benign lichenoid keratosis (BLK) [18, 19]; congenital lipomatous overgrowth, vascular malformations, epidermal nevi, scoliosis/skeletal and spinal (CLOVES; MIM # 612918) syndrome [6, 20]; Klippel-Trenaunay syndrome (KTS; MIM # 149000) [21]; and the related megalencephaly syndromes comprising megalencephaly-capillary malformation polymicrogyria syndrome (MCAP; previously/also known as macrocephaly-capillary malformation, MCM or macrocephaly-cutis marmorata telangiectatica congenita, MCMTC; MIM # 602501) [22], hemimegalencephaly (HMEG) [23] and dysplastic megalencephaly (DMEG) [24]; and venous malformation in the event of *PIK3CA* mutations [25].

In addition, other asymmetric overgrowth syndromes exist in the following: (1) the PI3K/AKT signaling pathway [1, 2] including Proteus syndrome (PS; MIM # 176920), caused by somatic activating mutations of *AKT1* [v-AKT murine thymoma viral oncogene homolog; *AKT1-E17K*] (on chromosome 14q32.33) [26]; hypoinsulinemic hypoglycaemia with hemihypertrophy (HIHGHH; MIM # 240900), caused by somatic activating mutations of *AKT2* (on chromosome 19q13.2) [27]; megalencephaly-polymicrogyria-polydactyly hydrocephalus syndrome (MPPH2; MIM # 615937), caused by somatic activating mutations of *AKT3* (on chromosome 19q43-q44) [28]; and (2) in the PI3K/PTEN signaling pathway [1, 2] including the Bannayan-Riley-Ruvalcaba syndrome (BRRS; MIM # 153480) [29] and the Cowden (Lhermitte-Duclos) syndrome 1 (CWS1; MIM # 153850), caused by mutations in the *PTEN* [phosphatase and tensin homolog] gene (on chromosome 10q23.31) [9,30].

Currently, no drugs have been approved for the treatment of these diseases, and surgery or symptomatic therapies are the only feasible interventions. With the development of genetic detection technologies, so far two critical genes, *PIK3CA* and *AKT*, have been identified with activating mutations in the PI3K/AKT pathway. The constitutive activation of *PI3K* and *AKT* (*AKT1* or 3) is causative for the initiation and progression of overgrowth syndromes [1–3, 6].

The PI3K/AKT signaling pathway plays a very important role in biological processes including cell growth and proliferation, metastasis, protein synthesis, angiogenesis, and survival [31–33]. Aberrant activation of this pathway has been associated with various types of cancers. Mutations of *PIK3CA* (6–35%) are more frequent in cancers as compared to *AKT1* mutations (0–8%) [34, 35]. Activating mutations of *PIK3CA* lead to increased PI3K activity, resulting in a higher output of PIP3 (phosphatidylinositol (3, 4, 5)-trisphosphate). Subsequently, PIP3 recruits AKT to plasma membrane where AKT is fully activated upon phosphorylation of T308 and S473 by PDK1 and mTORC2. Activating mutations of *AKT1* (e.g., *AKT1-E17K*) not only utilize PIP3 but also increase its affinity to PIP2 by more than 100-fold compared to wild-type *AKT1* [36]. Thus, both PIP2 and PIP3 apparently convert inactive confirmation of AKT to an active confirmation by binding to the PH domain and enhance the membrane recruitment and its activity. Once activated, AKT activates downstream targets (i.e., PRAS40), but suppresses TSC1/TSC2 activity [37–39]. As a critical node linking PI3K and mTOR pathways, AKT has become an ideal target for therapeutic intervention, and AKT inhibitors are being developed in various stages [40]. Thus, inhibition of AKT should be beneficial for patients with overgrowth syndromes driven by activating mutations of *PIK3CA* and *AKT*. Generation of mouse models to reflect the phenotypes of patients with overgrowth syndromes is challenging. So far, only mouse models with MCAP have been established, when activating *PIK3CA* mutations were introduced and accurately recapitulated several key human symptoms such as enlarged brain, cortical malformation, hydrocephalus, and epilepsy. Inhibition of PI3K activity in these mice, using PI3K inhibitors, alleviated some symptoms such as epilepsy [41]. Our previous studies showed that in primary PROS patient-derived cells, the PI3K pathway is overactive even in the absence of mitogens in culture, and their proliferation is PI3K-dependent for all mutations analyzed [11]: patients’ derived cells displayed a significant impairment of the proliferation rate upon treatment with PI3K inhibitors [11].

ARQ 092 is an orally bioavailable allosteric AKT inhibitor with high potency and selectivity. Both biochemical and cellular studies showed that ARQ 092 inhibited AKT activity through binding to its active and inactive forms. Cancer cell lines or patient-derived tumors harboring PIK3CA or *AKT1-E17K* mutations exhibited increased sensitivity to ARQ 092 treatment [42]. Somatic mutations of (a) *AKT1 E17K* have been associated with Proteus syndrome and a number of cancers [26, 43]; (b) *AKT2* with lipodystrophy and hypoglycaemia [27]; and *AKT3* with neuronal migration defects and hemimegalencephaly [28]. Targeting AKT permits the inhibition of the PI3K pathway closely downstream of this kinase but upstream of mTOR and circumvents the activation of additional pathways dependent on multiple classes and isoforms of PI3K kinases [44].

Herein, we report our findings on PROS patient-derived cells obtained from six affected individuals with extremely heterogeneous phenotypes (spanning from macrodactyly to MCAP) harboring different activating mutations of the *PIK3CA* gene at different frequencies in affected tissues. ARQ 092 showed higher anti-proliferative activity with lower cytotoxicity as compared to other PI3K inhibitors [e.g., mTOR inhibitors] [42, 45], thus indicating that (a) PROS-derived cells are dependent on AKT activity and (b) inhibition of mTORC1 does not represent a solid option in treating PROS patients. Our preclinical results suggest that ARQ 092 may be more effective, clinically, than other therapeutic options currently available, which show only a limited benefit.

## Subjects and methods

### Patients and clinical findings

#### Patient 1 [*hemihyperplasia multiple lipomatosis*, HHML]

**Fig. 1:**
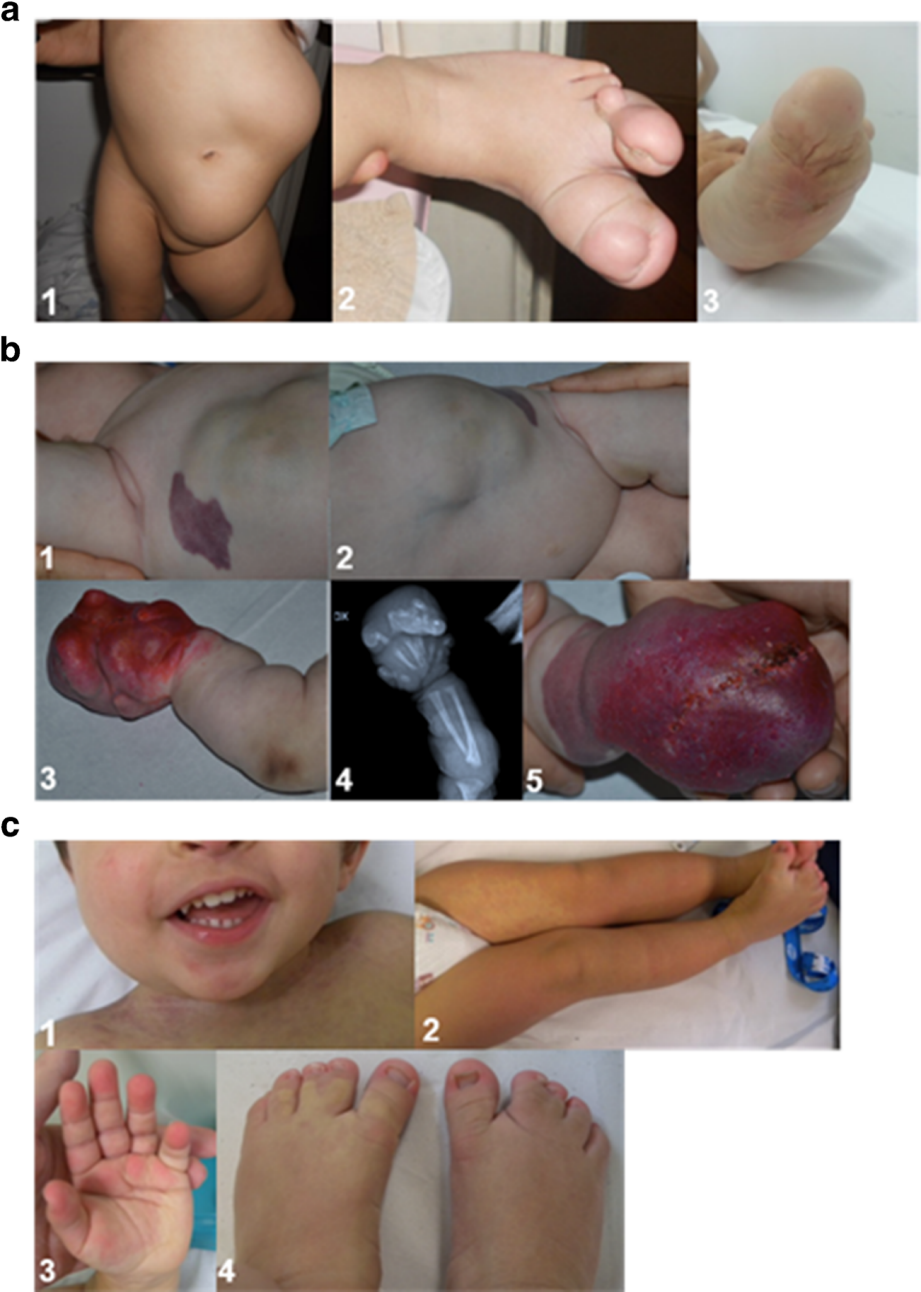
Spectrum of clinical features in patients with somatic *PIK3CA* mutations. **a** Patient 1: **1** frontal view showing the subcutaneous mass in the left abdominal region and the hypertrophy of her left leg [at age 1 year and 4/12 months]; **2** dorsal view of the left foot showing macrodactyly of the I and II toe with partial syndactyly between the 2nd and 3rd toe [at age 2 months]. **3** View of the sole showing the enlarged left foot [at age 5 years]. **b** Patient 2 [at age 1 year and 6/12 months]: **1**–**2** note the nodular mass involving the right side of the trunk; and **3** and **5** the gigantism and dysmorphisms of the right hand; **4** X-rays of the right hand showing exadactyly and dysmorphic features of the II, IV, and V metacarpal bones. **c** Patient 4 [at age 1 year and 2/12 months]: **1** note the facial asymmetry and **2**–**3** the vascular anomalies including diffuse capillary malformations and angiomas on the fingertips; and **4** the bilateral 2nd and 3rd toe syndactyly

This 6-year-old girl is the third child of a healthy 42-year-old woman and a non-consanguineous 52-year-old healthy man, whose family history was unremarkable. She was conceived naturally. She was born at term after a normal pregnancy: her fetal ultrasound scans were normal. Birth weight was 3.800 g (75th centile), length 51 cm (75th centile), and head circumference 36 cm (75th centile). At birth, macrodactyly of the I and II toe of the left foot with partial syndactyly between the 2nd and 3rd toes was recorded. Based on these clinical data, she was suspected to have Proteus syndrome. At the age of 1 month, a subcutaneous mass in her left abdominal region was observed. General physical examination at age 2 months showed that her weight was 5600 g (90th percentile), length 56 cm (50th percentile), and head circumference 38.5 cm (50th percentile). She presented macrodactyly of the I and II toes of the left foot with increased growth of the left leg and a subcutaneous mass in the left abdominal region; magnetic resonance imaging (MRI) of the abdomen revealed that the mass was compatible with a subcutaneous lipoma, which was later confirmed by histological examination of a sample of biopsied tissue. The girl was first referred to one of our institutions at age 4 months and followed up at age 9 months, 3 years and 10/12 months, and 5 years; she is still under follow-up at our institutions. Cognitive development is normal. During her last diagnostic work-up and follow-up controls, she underwent surgery for reduction of the abdominal mass and for removal of the first toe and transposition of the second toe to replace the first toe. Skin biopsies from the affected (and unaffected contralateral) regions were obtained during these procedures (Fig. 1a).

#### Patient 2 [congenital lipomatous overgrowth, vascular malformations, epidermal nevi, scoliosis/skeletal and spinal, CLOVES syndrome]

This 2-year-old boy is the second child of a healthy 27-year-old woman and a non-consanguineous 28-year-old healthy man. Their family history was unremarkable. He was conceived naturally and was born at term after an uncomplicated pregnancy: his fetal ultrasounds were normal. His birth weight was 3650 g (75th percentile), length 52 cm (75th percentile), and head circumference 33 cm (50th percentile). Postnatally, increased growth of the trunk, widespread cutaneous capillary malformations, and gigantism with exadactyly of the right hand became evident, suggestive for a complex lymphatic-arteriovenous overgrowth malformation. Upon monthly follow-up visits, weight and length growth were regular; however, subcutaneous masses (Fig. 1A, 1B) and increased growth of the hand became more evident (Fig. 1B: 3,5). His development was apparently normal. MRI and computerized tomographic (CT) scans revealed vascular malformations and vesicles within the masses noted in the trunk, with variable extension into the retroperitoneal and mediastinum regions; x-ray examination of the hands confirmed the skeletal exadactyly of the right hand; histological examination of the affected (and unaffected) tissues confirmed the initial hypothesis of a mixed lymphatic-arteriovenous malformation in the spectrum of CLOVES syndrome. Skin biopsies from the affected (and unaffected contralateral) regions were obtained during these procedures (Fig. 1b).

#### Patient 3 [*megalencephaly-capillary malformation polymicrogyria syndrome*, MCAP]

This 17-year-old boy was the only child born to non-consanguineous parents. His father and grandfather showed diffuse angiomatosis. He was conceived naturally. His fetal ultrasound scan showed IUGR. At birth, a diffuse capillary malformation involving the trunk and limbs was recorded. At age 3 months, he was noted to have left asymmetric overgrowth. During his follow-up visits, cardiac and abdominal ultrasound scans were repeatedly normal. Early developmental milestones were delayed, and at age 7 months, a brain MRI scan revealed focal hemimegalencephaly with right perisylvian polymicrogyria. From age 5 years, he started to manifest episodes of generalized tonic-clonic seizures, which proved to be refractory to antiepileptic therapy. He was severely cognitively impaired and developed an attention deficit disorder. On physical examination, at age 13 years and 8/12 months, his weight was 41.5 kg (10th–25th percentile), height 154.2 cm (10th–25th percentile), and head circumference 50.2 cm (2 SD < 3rd percentile). He had left asymmetric overgrowth, involving the face, trunk, and limbs (mainly the legs) with diffusely soft and thick irregularly marbled skin and prominent capillaries and veins on the trunk, abdomen, and limbs. His 2nd and 3rd left toes were significantly larger than the contralateral and showed proximal cutaneous syndactyly. Besides the increased growth, he had dysmorphic features including malar hypoplasia, long philtrum and high palate, and S-shaped scoliosis. Skin biopsies from the affected (and unaffected contralateral) skin regions were obtained.

#### Patient 4 [*megalencephaly-capillary malformation polymicrogyria syndrome*, MCAP]

This was the only child of healthy unrelated parents. His family history was unremarkable. Pregnancy was normal and repeated prenatal ultrasound evaluations were within normal limits. He was born at term through cesarean section because of breech presentation. Birth weight, length, and head circumference were at the 50th percentiles. Apgar scores were 10/10 at 5 and 10 min. Since birth multiple skin haemangiomas and diffuse capillary malformations were evident on the trunk, upper, and lower limbs. Mild facial asymmetry (right > left) was evident (Fig. 1c 1). He was first referred to one of our institutions at age 14 months. Physical growth and psychomotor development were normal; a relative macrocephaly was evident with no dysmorphic signs; no major malformations of internal organs were present. Cerebral MRI showed mild cranial asymmetry (right > left) and mildly ectopic cerebellar tonsils. Facial MRI confirmed right soft tissue hypertrophy. Physical examination and follow-up controls confirmed the facial asymmetry, the vascular malformation and the syndactyly of the toes (Fig. 1c). Skin biopsies from the affected (and unaffected contralateral) skin regions were obtained.

#### Patients 5 [*type 1 macrodactyly*] and 6 [*megalencephaly-capillary malformation polymicrogyria syndrome,* MCAP]

These patients were previously reported and are identifiable as patients 2 and 1, respectively [see reference 11]. Samples for this study were obtained from skin biopsies from the affected (and unaffected contralateral) skin regions.

### Patient recruitment

All patients (and/or their guardians) signed (or had previously signed [patients nos. 5 and 6 in reference 11] an informed consent approved by the local ethics committees to participate in this study and to authorize the publication of their clinical images. Blood and tissue samples were collected locally at the clinical centers and analyzed by means of the methods hereby reported.

### DNA extraction and Sanger sequencing

Genomic DNA was extracted from peripheral blood cells (PBCs) and tissue samples using the QIAamp Mini Kit (Qiagen, Hilden, Germany), according to the manufacturer’s instructions, and quantified on a Bio Spectrometer Plus (Eppendorf, Hamburg, Germany). The entire coding region of the *PIK3CA* gene was sequenced and analyzed according to the methods indicated in our previous report [11].

### Targeted deep sequencing

The Ion AmpliSeq Custom Panel of the 21 genes involved in the PI3K/AKT/mTOR pathway (i.e., *PIK3R1*, *PIK3R2*, *PIK3CA*, *PTEN*, *PDK1*,*PDK2*, *KRAS*, *AKT1*, *AKT2*, *AKT3*, *RICTOR*, *MAPKAP1*, *MLST8*, *MTOR*, *IRS1*, *GAB1*,*GAB2*, *THEM4*, *MAPK8I1*, *PTPN11*, and *RAPTOR*) was used according to our previous report [11]. Sequencing runs were performed on a Ion Torrent Personal Genome Machine (Life Technologies) using the Ion PGM Sequencing Hi-Q 200 Kit (Life Technologies), according to the manufacturer’s instructions [11].

### Alignment

Data analysis was performed using the Torrent Suite Software v5.0.5 (Life Technologies). Reads were aligned to the hg19 human reference genome from the UCSC Genome Browser (http://genome.ucsc.edu/) and to the BED file designed using Ion AmpliSeq Designer. Alignments were visually verified with the software Alamut® v2.8.0 (Interactive Bio software) (Fig. S1).

### Coverage analysis

The mean average read depth and the percentage of reads mapping on the ROI out of the total number of reads (reads on target) were calculated using the Coverage Analysis plugin (Torrent Suite v5.0.5 software, Life Technologies). For each sample, the percentage of ROI with a minimum coverage of 100× was calculated using the amplicon coverage matrix file (Table S1).

### Variant analysis

Variant calling was performed with the Variant Caller plugin configured with somatic high stringency parameters. Variants were annotated using the Ion Reporter 5.0 software (https://ionreporter.lifetechnologies.com/ir/). Common single nucleotide variants (minor allele frequency [MAF] > 5%), exonic synonymous variants, and intronic variants were removed from the analysis, while exonic non-synonymous, splice site, and loss-of-function variants were analyzed.

The sequence analysis software Alamut® v2.8.0 (Interactive Bio software) was used to interpret variants. Online databases, including dbSNP (database the single nucleotide polymorphism database), 1000 Genomes, ClinVar, EXAC (exome aggregation consortium), COSMIC (catalog of somatic mutations in cancer), ESP (exome sequencing project) were used. The pathogenicity prediction programs such as PolyPhen2, SIFT, Mutation Taster, and splice prediction programs were used to evaluate variants not previously described.

### Cell culture and reagents

Patient-derived primary fibroblasts were isolated according to our previous report (11) and grown in RPMI supplemented with 10% FBS, 100 IU/ml penicillin, 100 μg/ml streptomycin, and 1% L-glutamine in a humidified incubator at 37 °C and 5% CO_2_ avoiding confluence at any time. For phosphorylation studies, cells were grown in RPMI 10% fetal bovine serum or transferred to serum free medium for 6 h prior to testing the drugs. Wortmannin (10 μM) and rapamycin (100 nM) were purchased from Sigma-Aldrich (Poole, UK) and LY294002 (25 μM) from Selleckchem (Houston, TX). ARQ 092 was synthesized by ArQule, Inc. Woburn, MA.

### Cell counts/quantification of cell number

The number of primary cells was determined by counting. Cells were seeded into 24-well plates at 4.6 × 10^3^ cells/well 1 day before treatment. The end point of the cell culture experiments was collected before cells reached confluence. Supernatants (containing dead/floating cells) were collected and the remaining adherent cells detached by trypsin/EDTA (Sigma-Aldrich). Cell pellets were re-suspended in 1× PBS and 10 μl was mixed with an equal volume of 0.01% trypan blue solution. Trypan blue exclusion test was performed to determine viable (unstained, trypan blue negative cells) vs dead cells (stained, trypan blue positive cells). The percentages of dead cells were calculated. Cell numbers were counted with Countess II Automated Cell Counter (Life Technologies). Experiments were independently repeated at least three times.

### Cell proliferation assay (WST-1)

The anti-proliferative effect of ARQ 092 and of PI3K/AKT/mTOR inhibitors (wortmannin, LY294002, and rapamycin) was evaluated on PROS cells derived from the six patients enrolled in the study using the Cell Proliferation Reagent WST-1 (Roche, Mannheim, Germany) according to manufacturer’s instructions. Cells were seeded into 96-well plates at 3 × 10^3^ cells/well 1 day before treatment. The end point of the cell culture experiments was before reaching a confluent state. After 24, 48, 72, or 96 h of drug (or DMSO) exposure, 10 μl of the Cell Proliferation Reagent WST-1 was added to each well and incubated at 37 °C in a humidified incubator for 1 h. The absorbance was measured on a microplate reader (BioTek, Seattle, USA) at 450/655 nm. Each assay was performed in three replicates, and the experiment was repeated three times. The cell proliferation was calculated as the ratio of WST-1 absorbance of treated cells to WST-1 absorbance of control cells of the same experimental group.

### Immunoblot analysis

Immunoblotting analyses were performed according to the instructions of Cell Signaling Technology (Beverly, USA).

For each treatment, all cells grown on plates were collected and homogenized in 1× lysis buffer (50 mM Tris-HCl pH 7.4; 5 mM EDTA; 250 mM NaCl; 0.1% Triton X-100) supplemented with protease and phosphatase inhibitors (1 mM PMSF; 1.5 μM pepstatin A; 2 μM leupeptin; 10 μg/ml aprotinin, 5 mMNaF; 1 mM Na3VO4). Protein quantification was performed with MicroBCA™ Protein Assay Kit (Thermo Scientific, Cat#23235). Protein extracts of 20 μg from each sample were denatured in 5× Laemmli sample buffer. Proteins were separated in SDS-polyacrylamide gel and transferred to nitrocellulose membranes, using Trans-Blot® Turbo™ Mini Nitrocellulose Transfer (Biorad; Cat#1704158).

The blocking agents used were 5% BSA (bovin serum albumin; Sigma-Aldrich, Cat#A9418) for membranes incubated with anti-phospho antibodies and 5% nonfat dry milk (Blotting-Grade Blocker; Biorad, Cat#170-6404) for membranes incubated with anti-total antibodies.

Western blots were performed using primary antibodies at the dilution of 1:500 using 5% BSA for anti-phospho and 5% nonfat dry milk for anti-total, instead anti-ACTB was used at the dilution of 1:5000 with 5% nonfat dry milk. The antibodies used were as follows: polyclonal anti-ACTB (Sigma-Aldrich; Cat#A2066), monoclonal anti-phospho-AKT (Thr308) (Cell Signalling Technology; Cat#2965), polyclonal anti-phospho-AKT (Ser473) (Cell Signalling Technology; Cat#9271), polyclonal anti-AKT (Cell Signalling Technology; Cat#9272), monoclonal anti-phospho-AKT1S1 (Thr246) (Cell Signaling Technology; Cat#13175), monoclonal anti-AKT1S1 (Cell Signalling Technology; Cat#2691), polyclonal anti-phospho-RPS6KB1 (Ser371) (Cell Signalling Technology; Cat#9208), polyclonal anti-RPS6KB1 (Cell Signalling Technology; Cat#9202), monoclonal anti-phosho-RPS6 (Ser235/236) (Cell Signaling Technology; Cat#4858). Western blots were developed with the Clarity TM Western ECL substrate chemiluminescence reagent (Biorad, Uppsala, Sweden #1705061). The densitometric evaluation was performed by the ImageJ software.

### Statistical analysis

Statistical significance of the results was analyzed using the Student’s *t* test. A value of *P* < 0.05 was considered statistically significant.

#### Data availability

The datasets generated during and/or analyzed during the current study are not publicly available due to data and privacy protection considerations but may be available upon justified request.

## Results

### ARQ 092 inhibits AKT signaling in a dose- and time-dependent manner

In a previous study [11], we established primary fibroblast cells from two PROS patients and demonstrated that inhibition of PI3K activity suppressed cell proliferation and PI3K pathway. Although PI3K inhibitors in this study, wortmannin and LY294002, inhibited cell proliferation, the drug concentrations were very high [11]. Since AKT is a critical node between PI3K and mTOR in their signaling pathways, we hypothesized that specific inhibition of AKT could likely produce similar phenomena by using a potent selective allosteric AKT inhibitor, in this case ARQ 092 [42]. Four additional PROS patients were included in the current study. A targeted deep sequencing of 21 selected genes involved in the PI3K/AKT/mTOR pathway in blood and tissue/biopsy/cell culture samples from the six enrolled patients were performed (Table 1), with the methods previously described [11]. The mutant allele frequencies are from 11 to 57.1%, respectively.

**Table 1:** Phenotypes and frequency of mutations in the 6 PROS patients

PROS patients	Clinical phenotype	PIK3CA nucleotide change	PIK3CA amino acid change	Mutation frequency biopsy (%)	Mutation frequency cells (%)	Mutation frequency blood (%)	Mutation frequency saliva (%)
1	HHML	c.3140A > G	p.His1047Arg	57.1	57	absent	absent
2	CLOVES	c.3140A > G	p.His1047Arg	46.5	50	absent	absent
3	MCAP	c.2176G > A	p.Glu726Gly	37	37	N/A	N/A
4	MCAP	c.3139C > T	p.His1047Tyr	25	26	absent	19
5	Macrodactyly	c.3140A > G	p.His1047Arg	9	15	absent	N/A
6 LL	MCAP	c.241G > A	p.Glu81Lys	21.5	20	absent	42
6 RL	MCAP	c.241G > A	p.Glu81Lys	9	11	absent	

HHML: Hemihyperplasia multiple lipomatosis; CLOVES: congenital lipomatous overgrowth, vascular malformations, epidermal nevi, scoliosis/skeletal and spinal syndrome; MCAP megalencephaly-capillary malformation

**Fig. 2:**
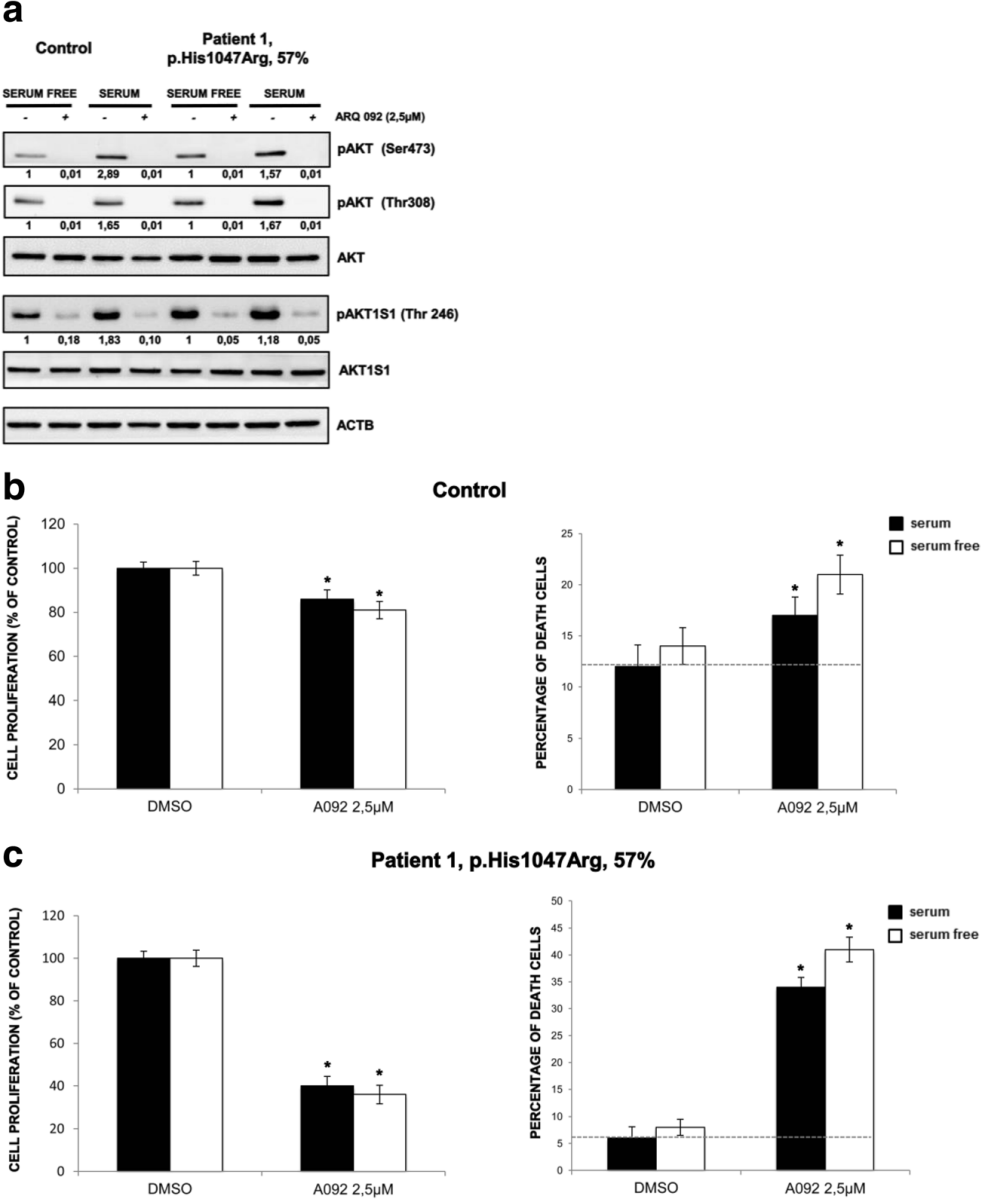
ARQ092 counteracts overactivation of PI3K signal pathway in both mutant and control cells. **a** Primary fibroblasts (from biopsies of a healthy control and patient 1) were treated with ARQ 092 (2.5 μM) in the presence (+) or absence (−) of serum and immunoblot analysis was performed to evaluate pAKT (Ser473), pAKT (Thr308), total AKT, pAKT1S1 (Thr246), and total AKT1S1. The reported values are the results of the densitometric analysis of the phosphorylated forms of the indicated proteins normalized against their total forms and the loading controls ACTB (arbitrary units; DMSO control = 1). **b** Primary fibroblasts obtained from biopsies of a healthy control and **c** of patient 1 were cultured with or without ARQ 092 2.5 μM in the presence or absence of serum for 72 h. The cell proliferation was determined using the WST-1 assay. Percentage of death cells was determined using Trypan Blue exclusion test. The results are presented from at least three independent experiments. Statistical analysis was performed using Student’s *t* tail test; *P < 0.05, which was considered statistically significant. The dotted lines correspond to the background control level of cell death detected in cells under standard conditions (DMSO)

**Fig. 3:**
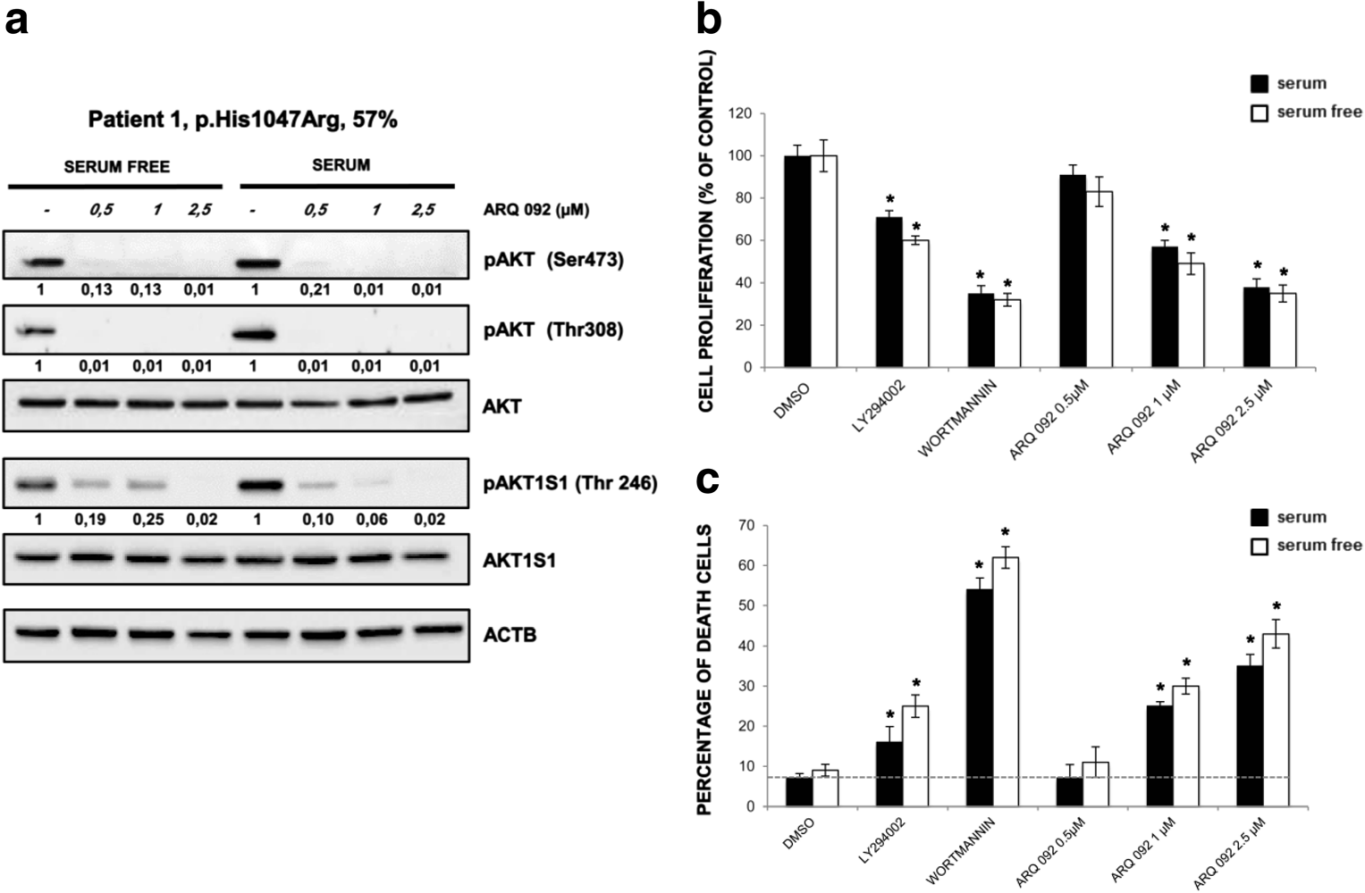
ARQ 092 decreased the level of pAKT and downstream target in a dose-dependent manner. **a** Primary fibroblasts (from biopsies of patient 1) were treated with ARQ 092 at 0.5, 1, and 2.5 μM in the presence or absence of serum for 72 h and Western blot analysis was performed to assess pAKT (Ser473), pAKT (Thr308), total AKT, pAKT1S1 (Thr246), total AKT1S1. The reported values are the results of the densitometric analysis of the phosphorylated forms of the indicated proteins normalized against their total forms and the loading controls ACTB (arbitrary units; DMSO control = 1). **b** Primary fibroblasts obtained from biopsies were cultured with or without LY294002 at 25 μM, wortmannin at 10 μM, or ARQ 092 at 0.5, 1, 2.5 μM in the presence or absence of serum for 72 h. The cell proliferation was determined using the WST-1 assay. **c** Percentage of death cells was determined using Trypan Blue exclusion test. The presented results are presented from at least three independent experiments. Statistical analysis was performed using Student’s *t* tail test; *P < 0.05, which was considered statistically significant. The dotted lines correspond to the background control level of cell death detected in cells under standard conditions (DMSO)

**Fig. 4:**
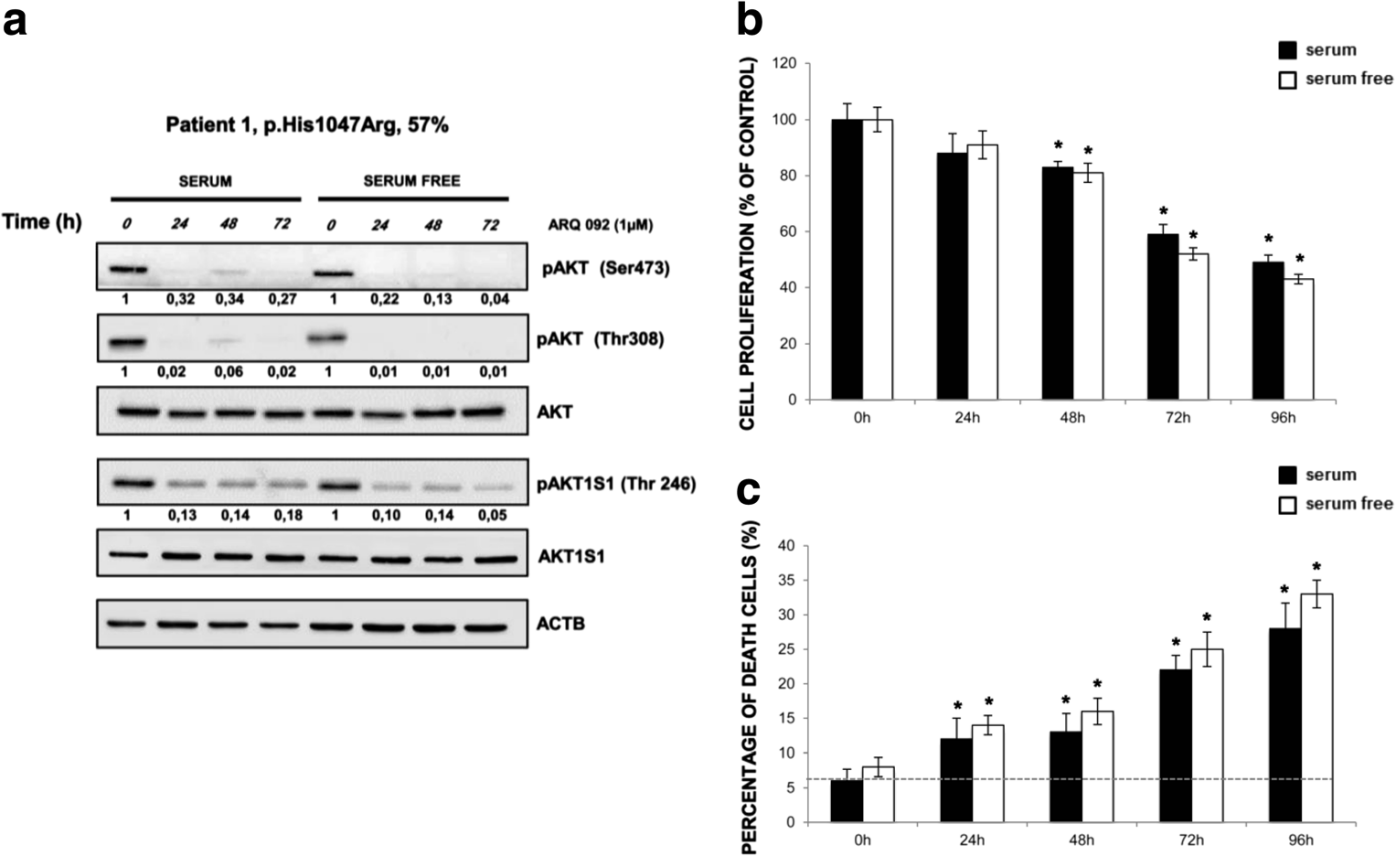
ARQ 092 decreased the level of pAKT and downstream target in a time-dependent manner. **a** Primary fibroblasts (from biopsies of patient 1) were treated with ARQ 092 at 1 μM in the presence or absence of serum for 24, 48, 72 h and Western blot analysis was performed to assess pAKT (Ser473), pAKT (Thr308), total AKT, pAKT1S1 (Thr246), total AKT1S1. The reported values are the results of the densitometric analysis of the phosphorylated forms of the indicated proteins normalized against their total forms and the loading controls ACTB (arbitrary units; DMSO control = 1). **b** The cell proliferation was determined at 0, 24, 48, 72, and 96 h using the WST-1 assay. **c** Percentage of death cells was determined using Trypan Blue exclusion test. The presented results are presented from at least three independent experiments. Statistical analysis was performed using Student’s *t* tail test; *P < 0.05, which was considered statistically significant. The dotted lines correspond to the background control level of cell death detected in cells under standard conditions (DMSO)

To evaluate the ability of ARQ 092 in counteracting the over activation of the PI3K/AKT/mTOR signaling, we treated primary fibroblasts derived from a healthy volunteer and from patient 1. Our results show that mutant fibroblasts had increased levels of phospho-AKT (pAKT) as compared to control cells when both were grown with or without serum (Fig. 2a). In both types of cells, ARQ 092 at 2.5 μM was able to significantly reduce the abundance of activated AKT, markedly in mutant cells, which was independent of culture conditions (Fig. 2a). Importantly, ARQ 092 blunted the AKT downstream signals, as shown by Western blot analysis of its direct phosphorylation target AKT1S1 (p-AKT1S1). Moreover, the results from evaluation of cell proliferation and cell death showed that control cells are less sensitive to ARQ 092 than the fibroblasts carrying PI3K mutation (Fig. 2b, c). These findings prompted us to extend our analysis by investigating whether a lower dose of ARQ 092 could significantly inhibit cell growth in vitro and reduce the extent of cell death. As shown in Fig. 3a, ARQ 092 at 0.5, 1, and 2.5 μM inhibited phosphorylation of AKT T308 and S473 and the levels of pAKT1S1 in a dose-dependent manner in primary fibroblasts from patient 1 (HHML) in the presence or absence of serum when compared to untreated primary fibroblasts, after 72 h of treatment. ARQ 092 did not change the levels of total AKT and AK1TS1. Anti-proliferative activity of ARQ 092 was further assessed at 0.5, 1, and 2.5 μM. ARQ 092 at 2.5 μM showed better or equivalent potency as LY294002 at 25 μM or wortmannin at 10 μM (Fig. 3b). Furthermore, the effect of ARQ 092 on cell death was evaluated as shown in Fig. 3c. Even at the highest concentration used (2.5 μM), ARQ 092 maintained lower cell death levels than wortmannin. Apparently, the presence or absence of serum did not significantly affect the activity of ARQ 092. Next, we determined the time-dependent response of AKT pathway towards ARQ 092. ARQ 092 at 1 μM apparently did not show a statistically significant difference in anti-proliferative effect compared to 2.5 μM ARQ 092 (Fig. 3b). In addition, based on ARQ 092 clinical study, ARQ 092 can reach C_max_ between 1 and 1.5 μM. It is rationale to use 1 μM for long-term treatment. Thus, we used ARQ 092 at 1 μM for long-term treatment. Primary fibroblasts were treated with ARQ 092 at 1 μM in the presence or absence of serum for 24, 48, 72, and 96 h. ARQ 092 reduced phosphorylated AKT (Ser473 and Thr308) and AKT1S1 after 24 h of treatment. ARQ 092 did not change the levels of total AKT and AKT1S1 (Fig. 4a). Anti-proliferative activity was observed at 72 and 96 h (Fig. 4b). Similarly, significantly increased cell death was observed after from 72 h (Fig. 4c). Taken together, these data demonstrated that ARQ 092 inhibited patient cell proliferation, accompanied with inhibition of AKT pathway.

### ARQ 092 inhibits AKT downstream signaling

**Fig. 5:**
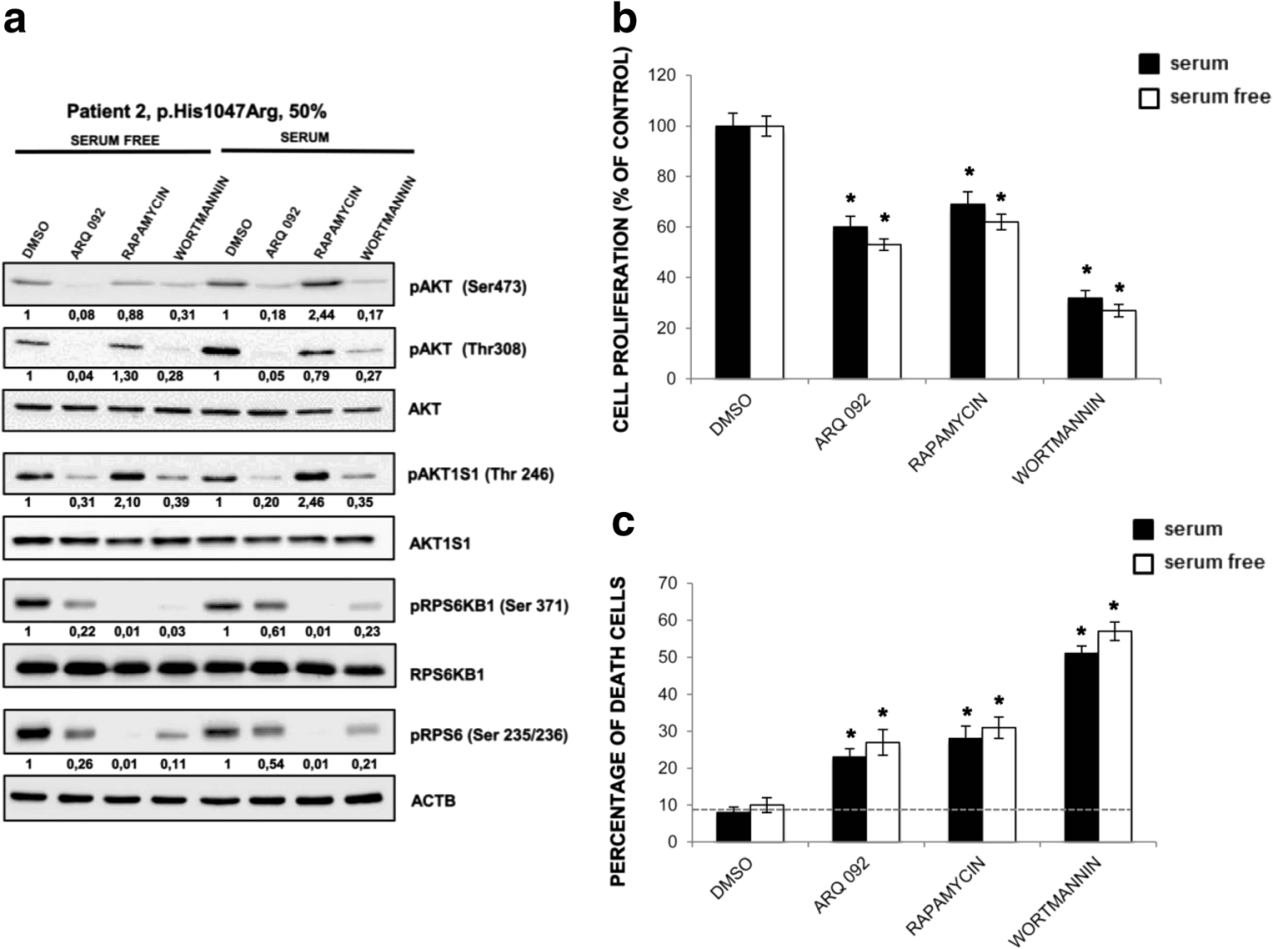
Effect of ARQ 092 is different from rapamycin and wortmannin in primary fibroblasts. Primary fibroblasts (from biopsies of patient 2) were incubated with ARQ 092 at 1 μM, rapamycin at 100 nM, or wortmannin at 10 μM in the presence or absence of serum for 72 h. **a** Western blot analysis was performed to assess pAKT (Ser473), pAKT1 (Thr308), total AKT, pAKT1S1 (Thr 246), total AKT1S1, pRPS6Kβ1 (Ser371), total RPS6Kβ1, pRPS6 (Ser235/236). The reported values are the results of the densitometric analysis of the phosphorylated forms of the indicated proteins normalized against their total forms and the loading controls ACTB (arbitrary units; DMSO control = 1). **b** The cell proliferation was determined at 72 h using the WST-1 assay. **c** Percentage of death cells was determined using Trypan Blue exclusion test. The presented results are presented from at least three independent experiments. Statistical analysis was performed using Student’s *t* tail test; *P < 0.05, which was considered statistically significant. The dotted lines correspond to the background control level of cell death detected in cells under standard conditions (DMSO)

In order to fully characterize the PI3K/AKT/mTOR cascade, in the primary fibroblasts of patient 2 (CLOVES) harboring the oncogenic mutation PIK3CA p.His1047Arg with 50% mutant allele frequency, we evaluated the phosphorylation status of AKT (Ser473 and Thr308) and its downstream target, pAKT1S1 (Thr246), pRPS6 (Ser235/236), pRPS6Kβ1 (Ser371) (Fig. 5a). ARQ 092 at 1 μM abolished phosphorylation of AKT in the presence or absence of serum, whereas mTORC inhibitor rapamycin at 100 nM did not inhibit AKT phosphorylation. This effect was comparable to wortmannin at 10 μM. Both ARQ 092 and wortmannin decreased pAKT1S1 levels in a similar degree. Furthermore, rapamycin could suppress mTOR downstream targets, RPS6KB1 and RPS6 more potently than ARQ 092 and wortmannin. As shown in Fig. 5b, after 72 h, both ARQ 092 at 1 μM and wortmannin showed anti-proliferative effect. Moreover, treatment of ARQ 092 showed an inhibition of proliferation more potent than rapamycin. ARQ 092 at 1 μM could be associated with lower level of toxicity than PI3K inhibitor but comparable to rapamycin after cell death were assessed (Fig. 5c).

**Fig. 6:**
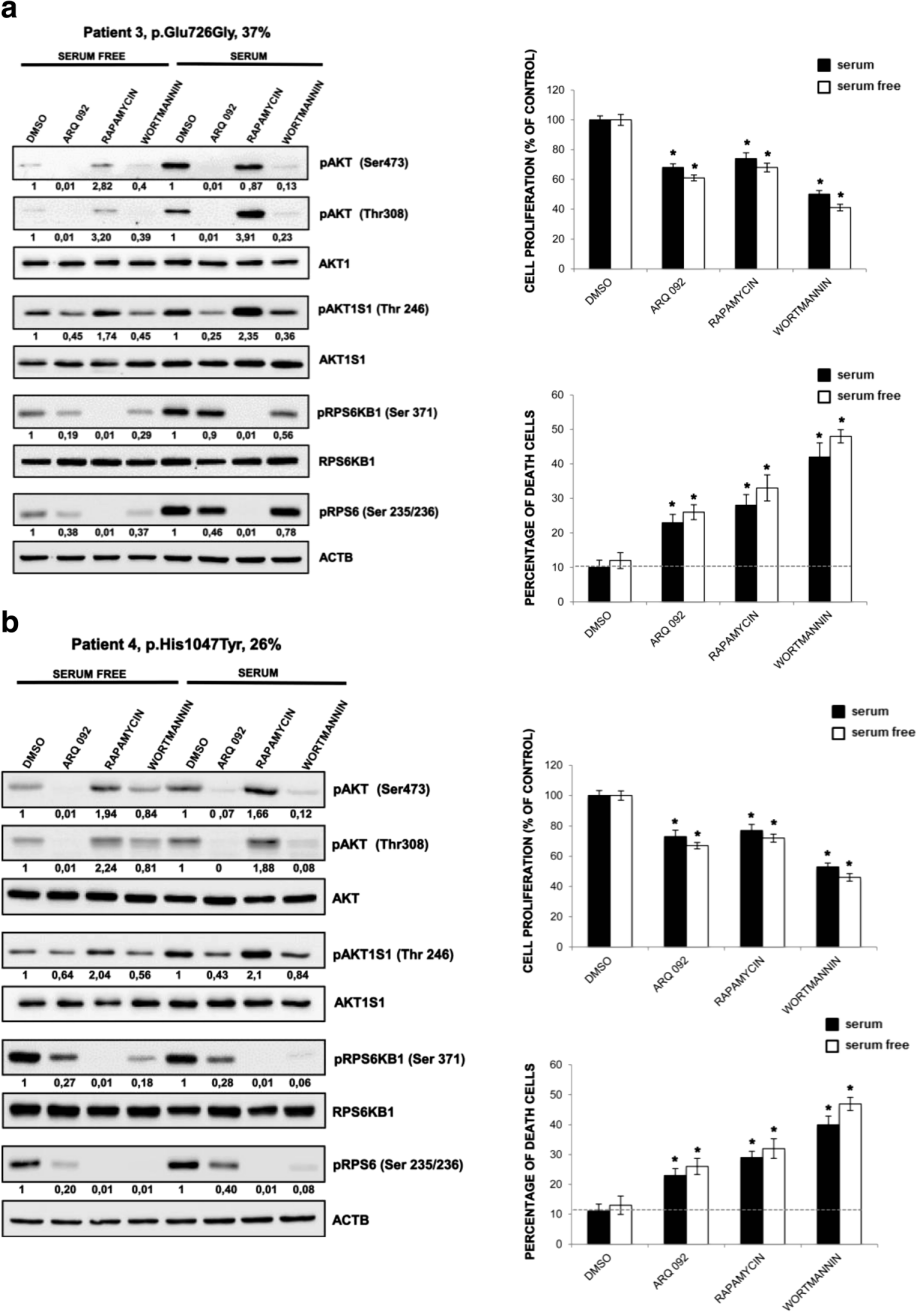
Effect of ARQ 092 on primary fibroblasts derived from patients 3 and 4. Primary fibroblasts from biopsies of patients 3 (**a**) and 4 (**b**) were incubated with ARQ 092 at 1 μM, rapamycin at 100 nM, or wortmannin at 10 μM in the presence or absence of serum for 72 h. **a** Western blot analysis was performed to assess phAKT (Ser473), pAKT (Thr308), total AKT, pAKT1S1 (Thr 246), total AKT1S1, pRPS6KB1 (Ser371), total RPS6KB1, pRPS6 (Ser235/236). The reported values are the results of the densitometric analysis of the phosphorylated forms of the indicated proteins normalized against their total forms and the loading controls ACTB (arbitrary units; DMSO control = 1). The cell proliferation was determined at 72 h using the WST-1 assay. Percentage of death cells was determined using Trypan Blue exclusion test. The presented results are presented from at least three independent experiments. Statistical analysis was performed using Student’s *t* tail test; *P < 0.05, which was considered statistically significant. The dotted lines correspond to the background control level of cell death detected in cells under standard conditions (DMSO)

**Fig. 7:**
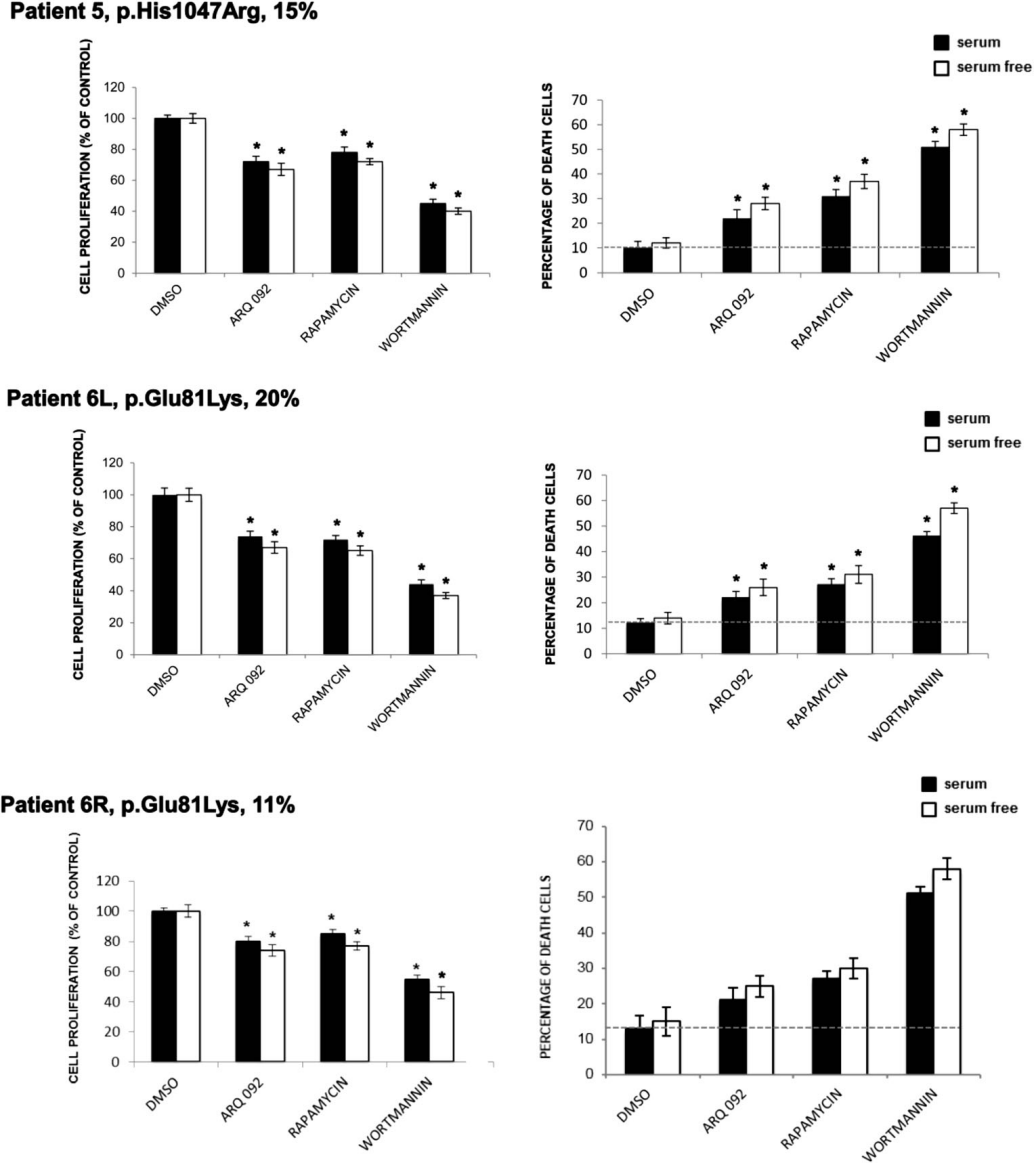
Anti-proliferative activity of ARQ 092 on primary fibroblasts derived from patients 5 and 6. Primary fibroblasts from biopsies of patients 5 and 6 were incubated with ARQ 092 at 1 μM, rapamycin at 100 nM, or wortmannin at 10 μM in the presence or absence of serum for 72 h. The cell proliferation was determined at 72 h using the WST-1 assay. Percentage of death cells was determined using Trypan Blue exclusion test. The presented results are presented from at least three independent experiments. Statistical analysis was performed using Student’s *t* tail test; *P < 0.05, which was considered statistically significant. The dotted lines correspond to the background control level of cell death detected in cells under standard conditions (DMSO)

To confirm that the above observations were the common phenomena, we further evaluated the effect of ARQ 092 in primary fibroblast cultures of patient 3 (MCAP) harboring the mutation PIK3CA p.Glu726Gly and patient 4 (MCAP) harboring the mutation PIK3CA p.His1047Tyr with mutant allele frequencies of 37 and 26%, respectively. As shown in Fig. 6a–b, ARQ 092 suppressed Akt signaling also in these cells independently of culture conditions and seems to be able to preferentially target mutated cells as shown by cell proliferation and cell death evaluation. In addition, results from primary fibroblast cultures of patient 5 (macrodactyly) (p.His1047Arg; 15%) and left leg (p.Glu81Lys; 20%) and right leg (p.Glu81Lys; 11%) of patient 6 (MCAP) showed that ARQ 092 induced an antiproliferative effect and associated to low index of toxicity as well in primary fibroblast cultures with lower mutant allele frequencies Fig. 7.

## Discussion and conclusions

*PIK3CA-related overgrowth spectrum* (PROS) represents a heterogeneous group of disorders, including congenital segmental overgrowth phenotypes with somatic *PIK3CA* mutations characterized by overlapping clinical features with variable tissue specificity. Some of these phenotypes are associated with pleiotropic and more severe manifestations [1–6]. *PIK3CA* mutations in PROS patients are identified in affected tissues at a variable frequency but are generally absent in blood and unaffected tissues [1, 2, 11]. The majority of somatic *PIK3CA* mutations, often strongly oncogenic, are frequently observed in several common human tumor types for which intensive efforts are under way to develop new drugs for use in common cancer therapy. *PIK3CA* alterations result in activated PI3K/AKT signaling [22, 32–34]. PROS patients might be regarded as appropriate candidates for enrolment in trials based on PI3K/AKT pathway inhibitors, considering also the “clean” cellular setting in which a unique driver, the PIK3CA mutation, is present versus the genomic heterogeneity seen in other PI3K/AKT driven diseases such as tumors. In this study, we evaluated the effects of ARQ 092, a novel AKT inhibitor, on primary fibroblast cultures from six PROS patients’ biopsies with identified and different PI3KCA activating mutations showing activation of the PI3K/AKT pathway. ARQ 092 is an orally bioavailable and highly selective AKT inhibitor currently under clinical development for the treatment of cancer and for patients affected by Proteus syndrome [42–45]. The usefulness of targeted therapies inhibiting the PI3K/AKT/mTOR pathway in PROS patients needs to be tested using patient-derived cells bearing the same frequency of PI3KCA mutations found in most patients. The use of patient-derived cells is necessary since very few mouse models for PROS are currently available [25]. We have previously shown that suppression of PI3K activity induced significant reduction of cell proliferation in three patient-derived fibroblasts [11]. Herein, we demonstrated that ARQ 092 markedly showed anti-proliferative activity in all of the primary fibroblasts derived from 6 patients (including samples obtained from the two patients previously tested for PI3K inhibition) in presence or absence of growth factors [11]. Interestingly, very high concentrations of PI3K inhibitors wortmannin and LY249002 (10 and 25 μM, respectively) exert equivalent antiproliferative effect. However, treatment with the PI3K inhibitor induced more cell death than ARQ 092, suggesting higher cytotoxic activity. Conversely, the mTOR inhibitor rapamycin at 100 nM exhibited weak anti-proliferative activity. A pathway analysis was performed to assess the changes in phosphorylation status of the AKT pathway. ARQ 092 was comparable or even more potent than PI3K inhibitors on AKT phosphorylation while rapamycin inhibited phosphorylation of ribosomal protein S6 kinase B1/S6 ribosomal protein, which indicates that ribosomal protein S6 kinase B1/S6 ribosomal protein activity may not be critical for patient-derived fibroblasts to proliferate. It has been demonstrated that ARQ 092 exerted minimal inhibition of phosphorylation of ribosomal protein S6, suggesting that inhibition of protein synthesis may not be required for the action of ARQ 092 [45].

As a critical node connecting the PI3K and mTOR pathways, targeting AKT has the advantage to inhibit the PI3K pathway and circumvent activation of additional pathways dependent on multiple classes and isoforms of PI3K kinases (classes I, II, and III). To date, the clinical anticancer efficacy of many PI3K inhibitors, apart from a few exceptions, is limited, because of an emerging resistance mechanism that determines reactivation of PI3K pathway or activation of complementary pro-survival pathways and significant toxicity for treated patients [46]. It is important to highlight here that the final goal in the treatment of overgrowth disorders, differently than cancer therapy, should be the reduction of the constitutive activation of PI3K/AKT axis together with lowering cytoxicity [45].

Although rapamycin (sirolimus) has been tested in a phase II trial against PROS (NCT02428296), accumulated studies have shown that Rapalogs (such as sirolimus) caused only partial inhibition of mTORC1 but had no effect on mTORC2, which induces persistent survival promoting AKT signaling. In addition, such weak inhibition of mTORC1 releases negative feedback leading to rebound activation of upstream signaling [46]. At this moment, lacking an approved therapy for this group of diseases, patients are receiving empirical therapies including mTOR inhibitors, but the ongoing testing of ARQ 092 in clinical trials may provide additional therapeutic intervention to this unmet medical need.

## Electronic supplementary material

Figure S1 Example of reads alignment views with Alamut software of the two index cases (patient 6 and 1). Alamut displays varying level of data detail depending on the zoom level. The position of the mismatch respect to the reference is delimited with blue bars and in the gray box frequency data and reads number are shown. (PDF 52 kb)

Table S1 (DOC 109 kb)

## Abbreviations

CLOVES: Congenital lipomatous overgrowth, vascular malformations, epidermal nevi, scoliosis/skeletal and spinal syndrome
COSMIC: Catalog of somatic mutations in cancer
dbSNP: Database the single nucleotide polymorphism database
DMEG: dysplastic megalencephaly
EXAC: Exome aggregation consortium
ESP: Exome sequencing project
FAO: Fibroadipose hyperplasia
HHML: Hemihyperplasia multiple lipomatosis
MCAP: Fibroadipose infiltrating lipomatosis, megalencephaly-capillary malformation
PIP3: Phosphatidylinositol (3,4,5)-trisphosphate
PROS: PIK3CA-related overgrowth spectrum
PS: Proteus syndrome
