# Supplementary material for: In vitro efficacy of ARQ 092, an allosteric AKT inhibitor, on primary fibroblast cells derived from patients with PIK3CA-related overgrowth spectrum (PROS)
Source: Neurogenetics. 2018 Mar 16;19(2):77–91. doi: 10.1007/s10048-018-0540-1 (PMC5956072; doi:10.1007/s10048-018-0540-1)
Supplement: Supplementary file 1 — Example of reads alignment views with Alamut software of the two index cases (patient 6 and 1). Alamut displays varying level of data detail depending on the zoom level. The position of the mismatch respect to the reference is delimited with blue bars and in the gray box frequency data and reads number are shown. (PDF 52 kb) [file 10048_2018_540_MOESM1_ESM.pdf]

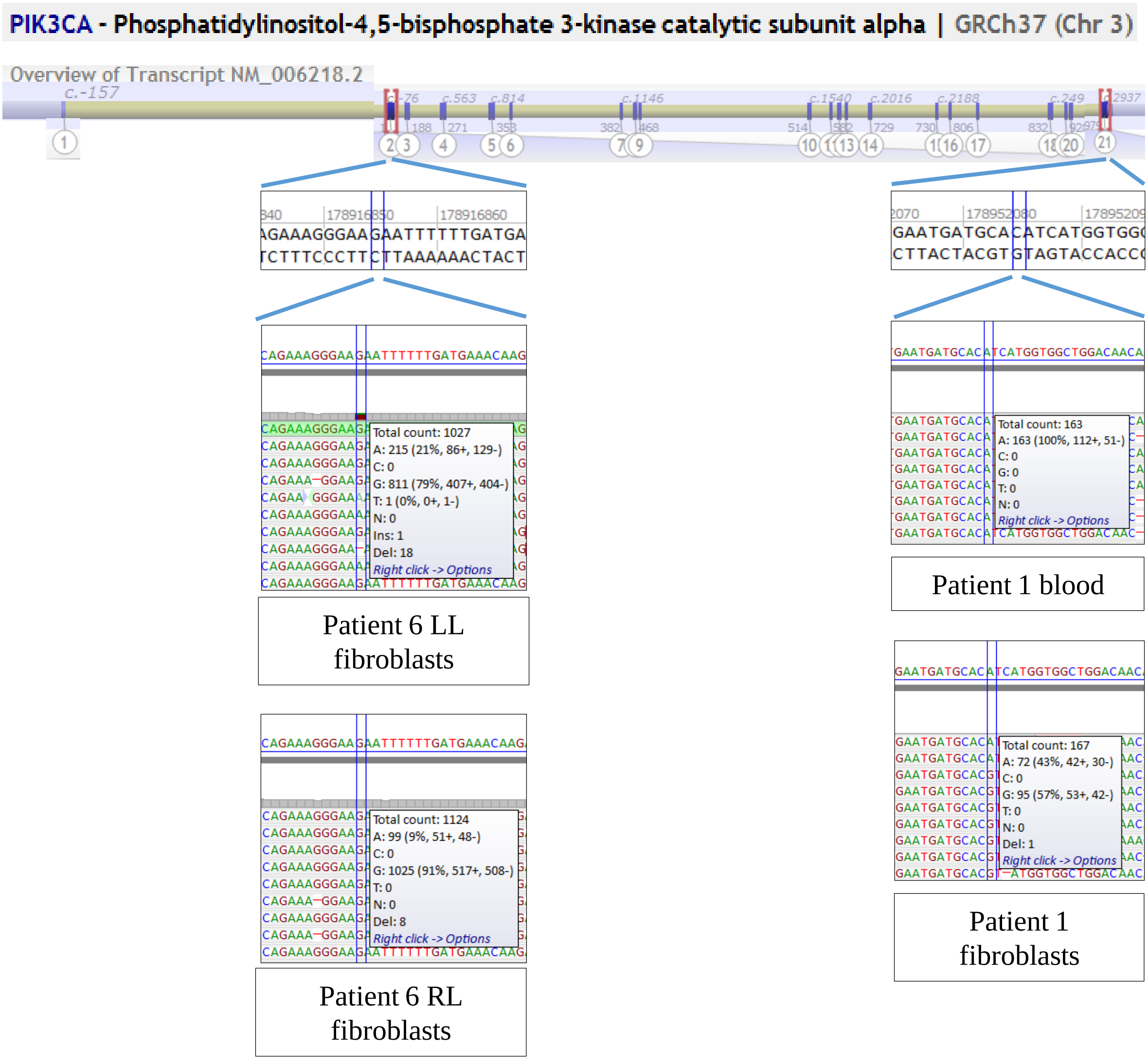
PIK3CA - Phosphatidylinositol-4,5-bisphosphate 3-kinase catalytic subunit alpha | GRCh37 (Chr 3)
Overview of Transcript NM_006218.2
178916850
178916860
178952080
CAGAAAGGGAAGAATTTTTGATGAAACAAG
Total count: 1027
A: 215 (21%, 86+, 129-)
C: 0
G: 811 (79%, 407+, 404-)
T: 1 (0%, 0+, 1-)
N: 0
Ins: 1
Del: 18
Right click -> Options
Patient 6 LL fibroblasts
Total count: 1124
A: 99 (9%, 51+, 48-)
C: 0
G: 1025 (91%, 517+, 508-)
T: 0
N: 0
Del: 8
Right click -> Options
Patient 6 RL fibroblasts
GAATGATGCACATCATGGTGGCTGGACAACA
Total count: 163
A: 163 (100%, 112+, 51-)
C: 0
G: 0
T: 0
N: 0
Right click -> Options
Patient 1 blood
Total count: 167
A: 72 (43%, 42+, 30-)
C: 0
G: 95 (57%, 53+, 42-)
T: 0
N: 0
Del: 1
Right click -> Options
Patient 1 fibroblasts
