# Supplementary material for: In vitro efficacy of ARQ 092, an allosteric AKT inhibitor, on primary fibroblast cells derived from patients with PIK3CA-related overgrowth spectrum (PROS)
Source: Neurogenetics. 2018 Mar 16;19(2):77–91. doi: 10.1007/s10048-018-0540-1 (PMC5956072; doi:10.1007/s10048-018-0540-1)
Supplement: Supplementary file 2 — (DOC 109 kb) [file 10048_2018_540_MOESM2_ESM.doc]

**Table S1**

Matrix table depicting the raw data as generated by the Ion PGM NGS sequencing instrument

| PROS Patients | 1  Fibroblasts | 1  Blood | 2  Biopsy | 2  Blood | 3  Fibroblasts | 4  Biopsy | 4  Blood | 5  Fibroblasts | 5  Blood | 6LL  Fibroblasts | 6RL  Fibroblasts |
| --- | --- | --- | --- | --- | --- | --- | --- | --- | --- | --- | --- |
|  |  |  |  |  |  |  |  |  |  |  |  |
| Total Reads | **649974** | **365087** | **604026** | **532239** | **820216** | **717515** | **1418109** | **1666507** | **138055** | **846565** | **863458** |
| % Reads on target | **67.28** | **83.68** | **85.56** | **82.88** | **67.79** | **85.69** | **85.28** | **96.54** | **96.35** | **96.62** | **96.59** |
| % Uniformity | **50.92** | **87.9** | **93.24** | **92.7** | **93.17** | **93.45** | **93.35** | **94.83** | **94.92** | **94.29** | **94.44** |
| Mean Read Lenght (bp) | **102** | **134** | **160** | **161** | **152** | **163** | **160** | **150** | **149** | **155** | **154** |
| Mean ROI Coverage* | **789.4** | **551.4** | **933** | **796** | **1004** | **1110** | **2183** | **2904.19** | **2401.06** | **1476.44** | **1505.51** |
| % ROI Covered>100X | **62.45** | **90.61** | **94.77** | **95.31** | **96.39** | **95.85** | **97.29** | **97.83** | **97.65** | **95.31** | **95.31** |
| N° Variants | **161** | **101** | **68** | **70** | **79** | **82** | **82** | **62** | **61** | **56** | **56** |

*average read per amplicon
